# aiDIVA – hybrid AI for rare disease diagnostics using evidence-based, machine learning and language models

**DOI:** 10.1038/s41525-026-00611-x

**Published:** 2026-08-29

**Authors:** Dominic Boceck, Lucia Laugwitz, Marc Sturm, Daniela Bezdan, Axel Gschwind, Tobias B. Haack, Stephan Ossowski

**Affiliations:** 1 Institute of Medical Genetics and Applied Genomics, University of Tübingen, Tübingen, Germany; 2 Institute for Bioinformatics and Medical Informatics (IBMI), University of Tübingen, Tübingen, Germany; 3 Neuropediatrics, General Pediatrics, Diabetology, Endocrinology and Social Pediatrics, University of Tübingen, University Hospital Tübingen, Tübingen, Germany; 4 Center for Rare Disease, University of Tübingen, Tübingen, Germany; 5 Center for Personalized Medicine, University of Tübingen, Tübingen, Germany; 6 Present Address: Institute of Pathology, University Hospital Ulm, Ulm, Germany

**Keywords:** Computational biology and bioinformatics, Diseases, Genetics

## Abstract

Genome sequencing enables accurate detection of genetic variants and is transforming rare disease diagnostics. While data generation is scalable, prioritization and clinical interpretation remain challenging, often requiring expert manual classification. AI-driven decision support systems are therefore needed to assist in causal variant identification or to fully automate large-scale re-analysis of unsolved cases. Existing tools often estimate variant impact on protein function, but few integrate genomic, phenotypic, and clinical annotation data for diagnosis. We present aiDIVA, an ensemble-AI combining statistical and machine learning models trained on genomic and phenotypic data to identify causal variants among tens of thousands per patient. aiDIVA applies a random forest model to classify pathogenicity and generates evidence-based scores for dominant and recessive diseases. These predictions are integrated with clinical metadata to prioritize the most likely causal variants. Large language models further refine and explain results. The aiDIVA-meta model consolidates all scores into a ranked list. aiDIVA-meta reported the causal variant among the top-3 candidates in 97.4% of a pre-training collected cohort with prior evidence in ClinVar or HGMD, and in 93.3% of a post-training collected cohort of previously unreported variants.

## Introduction

Advances in sequencing technologies have made genome sequencing (GS) feasible for clinical research and diagnostics. However, the unbiased detection of millions of germline variants per affected individual necessitates sophisticated prioritization strategies for the clinical interpretation of identified variants^1^. Despite recent improvements, the diagnostic rate for most rare genetic diseases (RD) remains below 40%, and numerous patients and their families continue their diagnostic odyssey^2,3^. Moreover, the growing complexity of genomic datasets and their analysis options may even exacerbate the bottleneck in final clinical evaluation and prolong the time to diagnosis and personalized treatment. Depending on the data background and clinical context, hundreds of variants may require manual evaluation despite rigorous bioinformatic pre-processing and filtering^4^.

Over the last decade, guidelines have been developed to assess the pathogenicity of genetic variants. The commonly used American College of Medical Genetics and Genomics (ACMG) guidelines^5^ categorize variants into five tiers: pathogenic, likely pathogenic, uncertain significance (VUS), likely benign, and benign. The classification process involves evaluating various types of evidence, including gene-level data, population data, computational predictions, functional data, and segregation information. Several tools that semi-automate the evaluation of ACMG criteria for specific subsets of disorders and genes are already available; however, their diagnostic accuracy is highly variable^6,7^. Furthermore, machine learning (ML) has been shown to improve pathogenicity prediction (ACMG criterion PP3) for small coding variants (e.g., CADD^8,9^, FATHMM-XF^10^, and REVEL^11^). These models are typically trained on variants with established ACMG classifications, e.g., from ClinVar^12^ or HGMD^13^. Usually, these models include a wide range of features, such as population allele frequency, evolutionary conservation, or structural effects on RNAs and proteins. However, studies have shown that model training is often biased by data circularity when the same training and evaluation data are repeatedly used across models^14^.

ML methods require several precomputed features generated by other tools or models, which are often incomplete (many pathogenicity predictors are only computed for coding variants) or outdated (some protein damage predictors are based on HG19 and must be lifted to newer assemblies). Extracting or updating features based on other functional impact models can therefore be tedious. Another shortcoming of most functional impact predictors is their focus on single-nucleotide variants (SNVs). In contrast, the classification of small in-frame insertions and deletions (indels) is often not included (e.g., AlphaMissense^15^, REVEL^11^). Only a few indel-specific algorithms are available, including CADD-Indel^8,9^, CAPICE-Indel^16^, FATHMM-indel^17^, and VEST-indel^18^. In clinical practice, most indel-specific tools do not perform sufficiently well to be included in a diagnostic workflow. Additionally, specialized tools, such as SpliceAI^19^, are required for the classification of splicing variants, and only recently, an AI model, PromoterAI, was published specifically targeting variants located in promoters^20^.

Despite continuous advancements in in silico prediction tools, the correct assignment of causal variants remains reliant on the correlation with detailed phenotypic information (ACMG criterion PP4). Tools that integrate phenotypic and molecular characteristics to pinpoint the causal variants underlying a given rare Mendelian disease are scarce. Among others, AI-MARRVEL^21^, Exomiser^22^, eDiVA^23^, DeepPVP^24^, Xrare^25^, and Lirical^26^ use different ML-based approaches to predict variant pathogenicity scores based on genomic and evolutionary features, which are then combined with Human Phenotype Ontology (HPO)^27^ terms and segregation information for causal variant prioritization. However, current approaches face several limitations. Most rely on precomputed pathogenicity scores that are often outdated or are not available for the current reference genome and certain variant classes, such as indels. Moreover, most methods rely on pre-computed HPO to disease gene maps and cannot screen the latest literature on novel disease gene associations. Finally, many methods were only evaluated on public datasets or small clinical cohorts, leading to uncertainty about their actual performance on novel disease cases in routine clinical diagnostics.

Recent advances in generative AI and the emergence of reliable large language models (LLMs) have spurred efforts to develop AI-assisted diagnostic methods^28,29^. These developments provide an opportunity to exploit the broad biomedical knowledge captured during model training to support clinical diagnostic decision-making. Training and fine-tuning of such models are facilitated by the vast amount of information found in clinical variant databases, ontologies, and publications. Recent studies have investigated the potential of LLMs to prioritize causal genes solely from phenotypic information. Kim et al.^30^ asked pre-trained LLMs to provide a list of the most suitable candidate genes for a disease phenotype described in the prompt. However, this approach identified the causal gene among the top 50 candidates in only 17.0% of test cases, leaving room for improvement. The Articulate Medical Intelligence Explorer (AMIE)^31^ is an LLM using clinical data (clinical history, physical examination, investigations, and procedures) to generate a differential diagnosis with good accuracy (top-10 accuracy 59.1%). Despite its good performance, AMIE is still limited by the inability to incorporate the patient’s genotype information or to prioritize causal variants for RD.

Here, we introduce aiDIVA, a tool that combines evidence-based and ML-based pathogenicity classifiers with large language models such as GPT-4o^32^, Llama^33^, or Mistral^34^. Compared to existing methods, our ensemble approach enables integration of genomic, evolutionary, clinical, phenotypic, and inheritance information from a broad range of sources, including the patient’s clinical data and genotypes, annotations from a wide range of genomic and clinical databases, HPO-gene maps, and the current literature to rank the most likely causal variants and to provide reasoning for suggested diagnoses. Moreover, other than competing methods, aiDIVA features a specialized model for in-frame indels.

aiDIVA was trained and tested using more than 150,000 known benign or pathogenic variants, as well as a large in-house cohort of cases diagnosed by clinical experts. We assessed aiDIVA using a benchmark set of more than 3000 RD cases curated in accredited routine diagnostics, identifying the causal variant among the top-3 ranked candidates in 97.4% of these cases with existing ClinVar or HGMD evidence. In addition, we evaluated aiDIVA on an independent cohort of 1014 cases diagnosed in our hospital after model training, whose causal variants were absent from ClinVar and HGMD at the time of training, achieving a top-3 sensitivity of 93.3% while minimizing circularity in benchmarking. Finally, we performed a comprehensive comparison with AI-MARRVEL, Exomiser, Lirical, and Xrare, demonstrating the superior performance of aiDIVA’s ensemble approach in routine clinical diagnostics.

## Results

### Pathogenicity classification and ranking of most-likely causal variants using aiDIVA

**Fig. 1:**
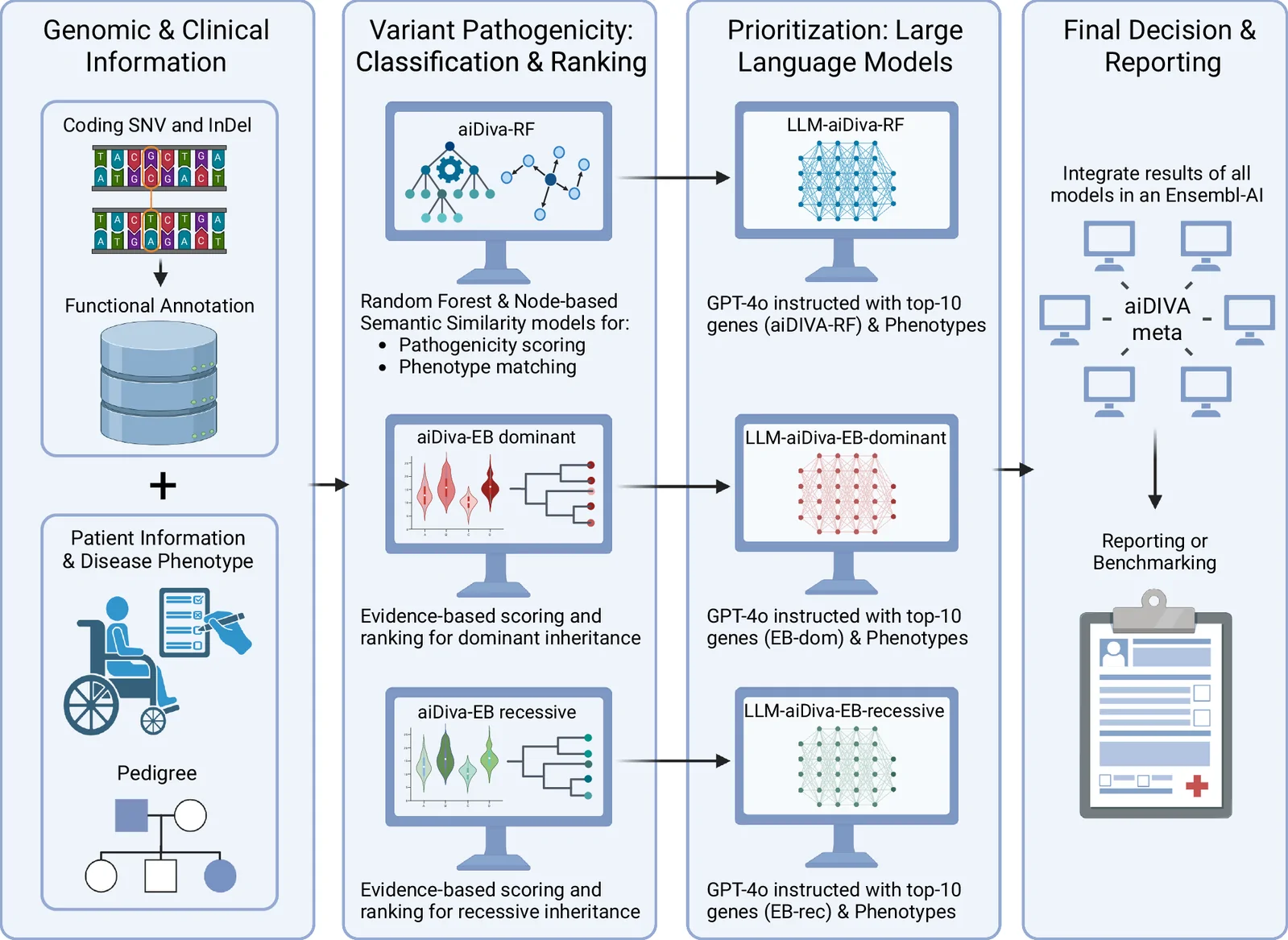
Workflow of the aiDIVA pipeline. First, three different variant rankings are created (aiDIVA-RF, aiDIVA-EB-dominant, and aiDIVA-EB-recessive). These different rankings are used as a basis for a refinement with LLMs (LLM-aiDIVA-RF, LLM-aiDIVA-EB-dominant, and LLM-aiDIVA-EB-recessive). Finally, the rankings and refinements are combined using a random forest model (aiDIVA-meta), resulting in a final ranking of causal variant candidates reported to the clinical expert. Created in BioRender. Ossowski, S. (2026) https://BioRender.com/wc5hp11.

We present benchmarking results for aiDIVA, a newly developed AI platform that integrates statistical models, ML classifiers, and LLMs to classify variant pathogenicity and prioritize causal variants in RD cases (Fig. 1). aiDIVA comprises two complementary models for pathogenicity scoring: The first component is an evidence-based model which leverages curated information from the scientific literature, established disease-associated variant databases such as ClinVar, HGMD, and OMIM, and gene-phenotype matching (Table 1). This model is loosely based on the ACMG guidelines and supports separate statistical frameworks for dominant and recessive Mendelian inheritance, implemented as aiDIVA-EB-dominant and aiDIVA-EB-recessive, respectively. The second component (aiDIVA-RF, Fig. 1 and Supplementary Fig. 1) is based on an RF classifier that integrates a broad range of genomic and clinical biomarkers. This model not only performs pathogenicity classification but also incorporates phenotype-driven, disease-similarity-based ranking to identify the most likely causal variants.

**Table 1:** Scoring criteria for the dominant and recessive models

Criterion	points (dominant)	points (recessive)
HPO term matches between patient and variant gene	1.0 + √(#matches) if there are matches	1.0 + √(#matches) if there are matches
OMIM gene	1.0 if in OMIM gene	1.0 if in OMIM gene
gnomAD allele frequency (overall)	1.0 if not in gnomAD 0.5 if below 0.01%	1.0 if not in gnomAD 0.5 if below 0.01%
HGMD pathogenicity	0.5 if flagged as pathogenic 0.3 if flagged as likely pathogenic	0.5 if flagged as pathogenic 0.3 if flagged as likely pathogenic
ClinVar pathogenicity	1.0 if flagged as pathogenic 0.5 if flagged as likely pathogenic	1.0 if flagged as pathogenic 0.5 if flagged as likely pathogenic
Conservedness (pyloP, 100 way)	0.3 if bigger than 1.6	0.3 if bigger than 1.6
Gene gnomAD o/e LoF	0.5 if below 0.1	0.5 if below 0.1
Gene inheritance mode*	0.5 if AD or XLD	0.5 if AR or XLR
In-house database count (heterozygous)*	1.0 if below 3 0.5 if below 6	-
Genotype*	-	1.0 if homozygous or two heterozygous hits in gene

*Most criteria or points are identical for the recessive and the dominant models. Only criteria marked with an asterisk differ between the models.

The top 10 candidates of each of the described models were then submitted to the LLM GPT-4o for inference of genotype-phenotype associations. Finally, the information from all models was transformed into features for the aiDIVA-meta model.

All models and competing approaches were benchmarked using 3041 RD cases previously solved by genetics experts without using AI.

### Cohort characteristics

The in-house cohort for benchmarking aiDIVA and competing classification models included 3041 NGS datasets from affected individuals with RDs, including 1975 ES and 1066 GS datasets. This cohort comprised individuals presumed to be affected by monogenic disorders, each harboring either a single pathogenic variant (homozygous or heterozygous) or two compound heterozygous pathogenic variants in the corresponding disease-associated gene.

Cases with causal copy number variants (CNVs) or other types of structural variants (SVs) were excluded. NGS data processing, including alignment, variant calling, and variant annotation, was performed using the megSAP pipeline^1,35^. Causal variants were initially identified by genetics experts for diagnostic purposes at our institution using the clinical decision support system GSvar^35^.

Available clinical data included the disease group, observed disease phenotypes characterized by HPO terms, age at diagnosis, and sex. The cohort included 1571 female and 1470 male individuals. The age at diagnosis ranged from newborn to a maximum of 91 years (mean: 26.54 years, median: 21 years). The largest disease groups were mental, behavioral, or neurodevelopmental disorders (*n* = 983); diseases of the visual system (*n* = 597); diseases of the nervous system (*n* = 587); neoplasms (*n* = 301); endocrine, nutritional, or metabolic diseases (*n* = 115); developmental anomalies (*n* = 107); and diseases of the musculoskeletal system or connective tissue (*n* = 90), as shown in Supplementary Fig. 2.

### Pathogenicity scoring

**Fig. 2:**
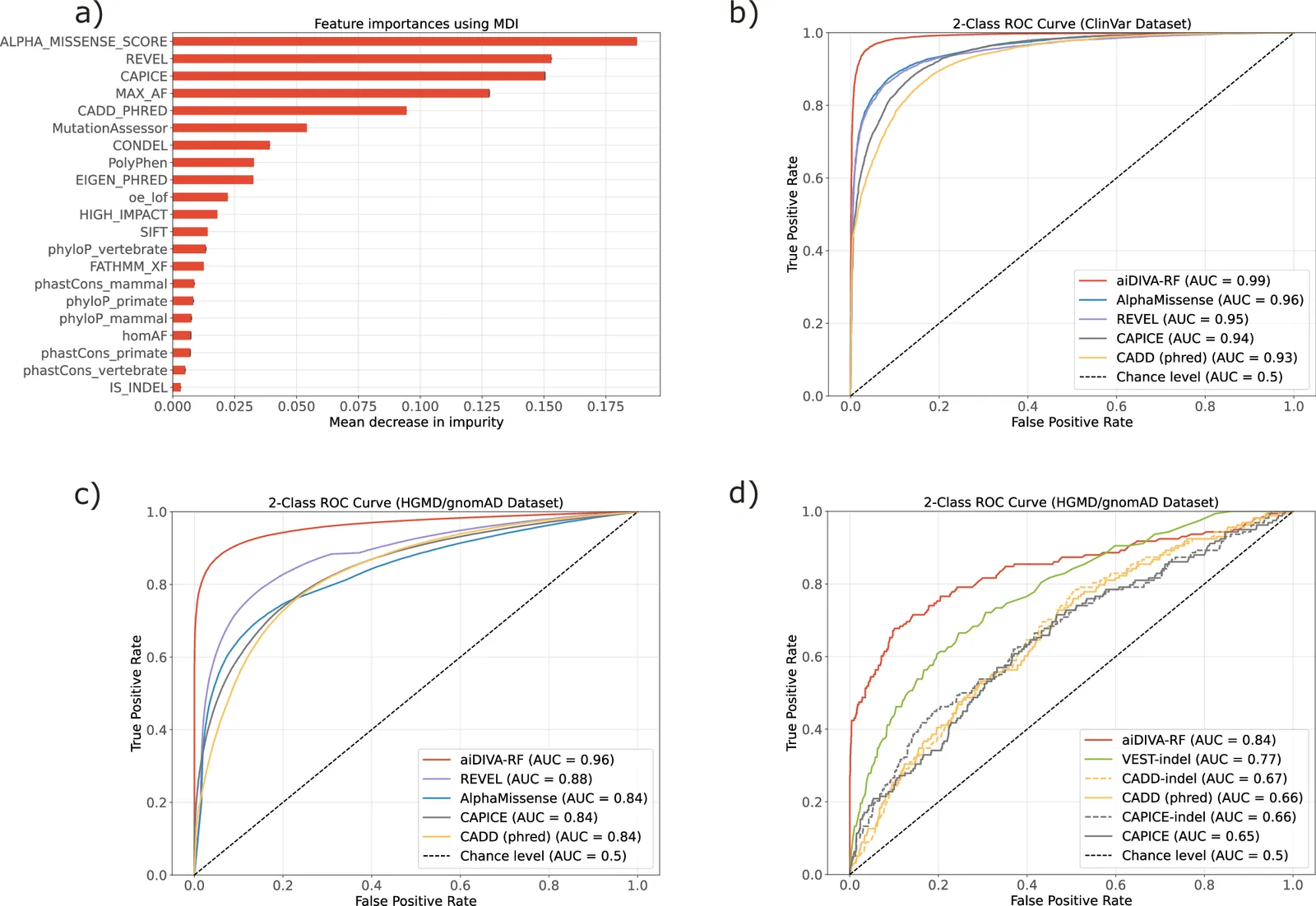
Pathogenicity scoring. **a** Feature importances of the aiDIVA-RF pathogenicity model measured with the mean decrease in impurity (MDI) **b** aiDIVA-RF pathogenicity prediction performance on the ClinVar test set. **c** aiDIVA-RF pathogenicity prediction performance on an independent HGMD/gnomAD test set. **d** Comparison between aiDIVA-RF pathogenicity model and three InDel-specific pathogenicity scores.

We trained the RF model aiDIVA-RF using 90,400 pathogenic and 65,202 benign variants from ClinVar^12^ and 21 genomic features (Supplementary Table 2). Feature importance, quantified as the mean decrease in impurity, is shown in Fig. 2a. Established pathogenicity estimators (e.g., AlphaMissense^15^ and REVEL^11^), population allele frequency (gnomAD^36^), evolutionary conservation (e.g., phyloP^37^ and phastCons^38^), and predicted functional impact on proteins (i.e., the HIGH impact class of VEP^39^) were among the most influential features contributing to accurate pathogenicity classification.

We compared the performance of aiDIVA-RF for SNV classification with other pathogenicity or functional impact classifiers, including AlphaMissense, REVEL, CAPICE, and CADD. Figure 2b illustrates the performance of the models on the ClinVar test set using only missense variants (10,044 pathogenic and 7245 benign variants) with the respective receiver operating characteristic (ROC) area under the curve (AUC). To test the model´s robustness and generalizability, we conducted additional benchmarking using an independent validation dataset (Fig. 2c), which included 103,000 pathogenic missense variants sourced from HGMD (not in ClinVar) and 106,975 presumed benign missense variants derived from gnomAD (see “Methods”). In both benchmarking analyses, aiDIVA-RF outperformed all competing methods. Specifically, when compared to AlphaMissense, aiDIVA-RF reached an ROC AUC of 0.99 versus 0.96 on the ClinVar test set and an ROC AUC of 0.96 versus 0.84 on the HGMD/gnomAD test set.

aiDIVA-RF incorporates a specialized model for in-frame indels, which simulates the impact of indels using a cluster of adjacent SNVs (see “Methods”). The performance of the indel pathogenicity classification could only be compared to CADD-indel, CAPICE-indel, and VEST-indel, as REVEL and AlphaMissense do not support indels. On the independent HGMD/gnomAD benchmark set containing only in-frame indels (3597 pathogenic and 3397 presumed benign variants), aiDIVA-RF demonstrated superior performance, achieving a ROC AUC of 0.84 (Fig. 2d).

### Benchmarking causal variant prioritization

**Fig. 3:**
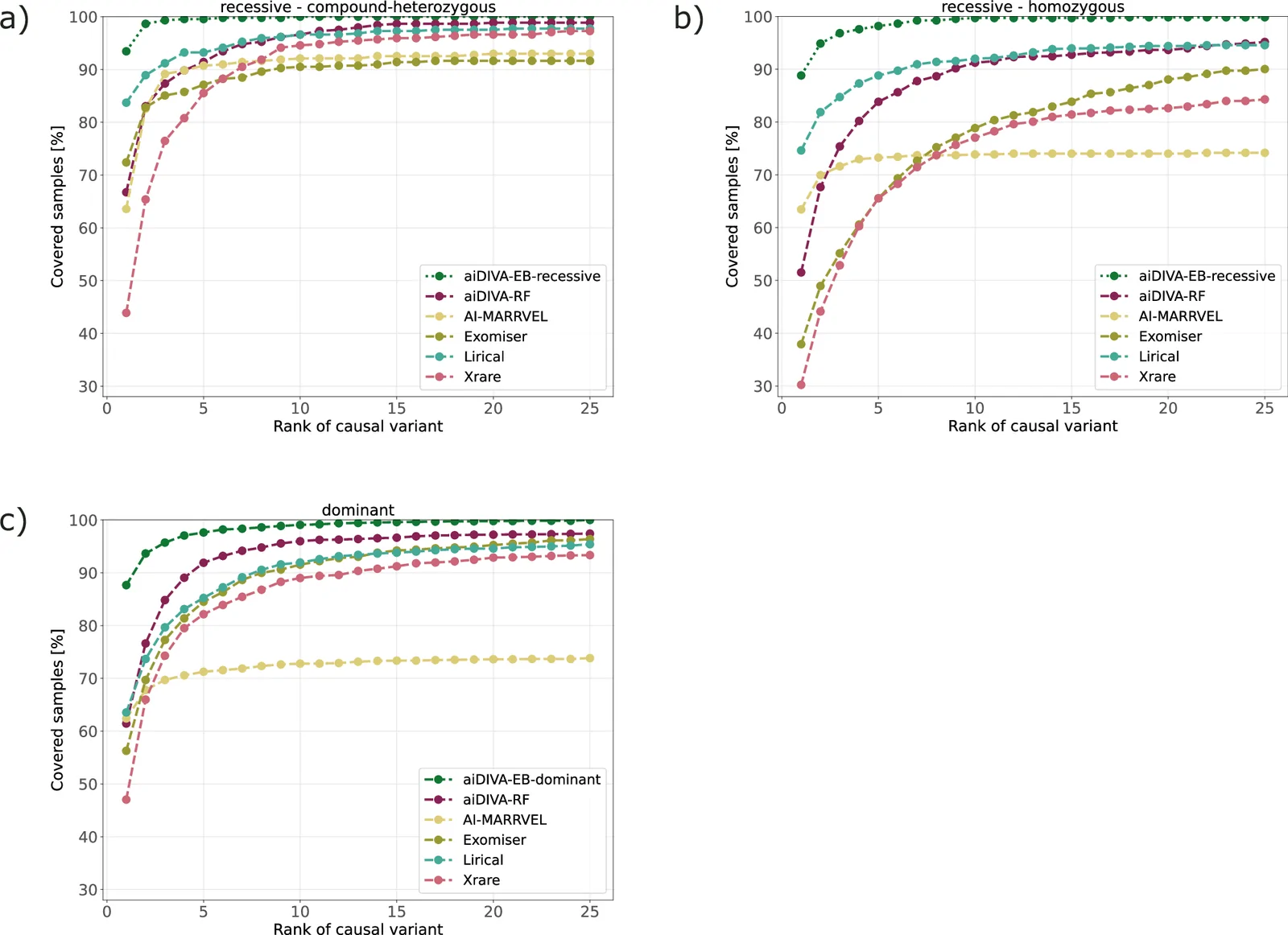
Diagnostic rank analysis comparison between the aiDIVA-RF ranking, the aiDIVA-EB-dominant/aiDIVA-EB-recessive ranking, and four competing tools (AI-MARRVEL, Exomiser, Lirical, and Xrare). We separated the results into three subsets based on the inheritance mode of the causal variants. **a** Recessive-compound-heterozygous subset of our benchmark set (442 cases), **b** recessive-homozygous subset (662 cases), **c** dominant subset (1937 cases).

aiDIVA provides two models for causal variant prioritization, which involve ranking the variants based on the likelihood of causing the genetic disease in the affected individual (aiDIVA-RF and aiDIVA-EB). A comparison of both approaches against AI-MARRVEL, Exomiser, Lirical, and Xrare on a benchmark dataset of 3041 expert-solved RD cases revealed the percentage of cases in which the models placed the correct variant on rank 1, up to rank 2, up to rank 3, and so on (Fig. 3).

The benchmark set was split according to the mode of inheritance into the subsets compound heterozygous (Fig. 3a), recessive (Fig. 3b), and dominant (Fig. 3c). aiDIVA-EB outperformed the other models in all inheritance modes, with approx. 90% of causal variants placed on rank 1. However, this level of performance is only anticipated in cases involving variants that have already been documented in at least one clinical database (hence referred to as the “evidence model”). aiDIVA-RF, on the other hand, does not explicitly incorporate information from ClinVar, HGMD, or similar clinical variant databases as features. aiDIVA-RF achieved superior performance compared to all competing methods, except for Lirical, which achieves slightly better performance for recessive cases, but worse performance for dominant inheritance. The performance gains of aiDIVA were most notable for dominant inheritance (Fig. 3c) and least evident for compound heterozygous traits (Fig. 3a). AI-MARRVEL exhibits an unusual performance profile, achieving relatively high accuracy at the top-1 ranking level (50%–60%), but showing an early plateau at a comparatively low maximum (e.g., 73% for dominant disorders), suggesting limited sensitivity in broader candidate prioritization. Interestingly, Exomiser and Xrare, two methods published years before AI-MARRVEL, still performed better than AI-MARRVEL for dominant and recessive cases when considering the top-20 ranked variants.

### Refining the causal variant ranking using large language models

aiDIVA uses the LLM GPT-4o^30^ to select and explain the three most likely causal variant candidates out of the 10 highest-ranked variants of each preceding model (aiDIVA-RF, aiDIVA-EB-dominant and aiDIVA-EB-recessive, Fig. 1). The prompt for GPT-4o included affected genes, disease phenotypes (encoded as HPO terms), age at diagnosis, and sex (see “Methods”). We examined the LLM´s inference results using aiDIVA-RF rankings as input. Specifically, we focused on the 2890 (out of 3041) benchmark cases in which the causal variant was ranked within the top 10 by aiDIVA-RF. This subset was further stratified into two groups: an “easy” subset, comprising 2528 cases where aiDIVA-RF ranked the causal variant within the top 3, and a “difficult” subset, consisting of the remaining 362 cases where the causal variant was ranked lower than third (Supplementary Fig. 3a, pie chart).

Initially, we tested a prompt version not specifying the individual variants affecting the top-10 genes. This approach yielded generally robust results, with rankings either improving or being stable in most cases. Notably, within the difficult subset, the ranking of the causal gene improved substantially, with 82.60% of cases (299 out of 362) now appearing in the top 3 reported by the LLM (Supplementary Fig. 3a, right bar). However, in the easy subset, a decline in performance was observed in 7.63% of cases (193 out of 2528), where the LLM no longer ranked the causal gene within the top 3 (Supplementary Fig. 3a, left bar). We hypothesized that (1) the reduced performance of the LLM in seemingly easy cases may be attributable to insufficient input information, and (2) cases with worsened rankings could be affected by limited reproducibility of LLM outputs.

We tested the reproducibility hypothesis by re-running the exact same prompt for two groups of cases from the “easy” group: (1) cases showing the correct gene among the top-3, and (2) cases with declined ranking not showing the correct gene in the top-3 as answer to the first prompt (Supplementary Fig. 3b). While the results in group 1 remained almost the same in the second prompting of GPT-4o, with only one case worsening, the answers in group 2 (declined ranking in first answer) improved in 27.46% of the cases (53/193), now reporting the correct variants among the top-3 as answers to the repeated identical prompt (Supplementary Fig. 3b, first bar).

We furthermore tested the general reproducibility of LLM responses by repeating prompts 5 times in 200 randomly selected cases from the easy and difficult groups. We observed that in 87.0% (82.0%) of easy (difficult) cases, all 5 repetitions recovered the correct gene in the top-3. In 7% (13%), the model reproducibly failed to report the correct gene in all repetitions. Only in 6.0% (5.0%) the responses were inconsistent, failing to report the correct gene in 1–4 out of 5 repetitions (for details see Supplementary Text and Supplementary Figs. 5 and 6). These findings support previous reports that repeating prompts can measure and potentially improve the reliability of a model^40^.

Furthermore, we tested the inclusion of additional information in the prompt, namely, the VEP impact (high, medium, or low) of each variant on the candidate genes, using the same two subgroups (improved/stable vs. declined ranking) of the “easy” cases. Inclusion of the variant effects in the prompt’s candidate gene list led to a strong performance gain in the declined-ranking group 2, with 40.93% of causal variants (79/193) now among the top-3 (Supplementary Fig. 3b, second bar). The Inclusion of variant zygosity led to an additional mild improvement to 45.6% causal variants (88/193) ranking among the top-3 (including the improvement from variant impact), while rankings in group 1 remained stable (Supplementary Fig. 3b, third bar).

In summary, prompt engineering led to an improved variant prioritization, with approximately 5% more causal variants found among the top-3 ranked genes compared to using aiDIVA-RF only.

### Diagnostic variant ranking using the aiDIVA-meta model

**Fig. 4:**
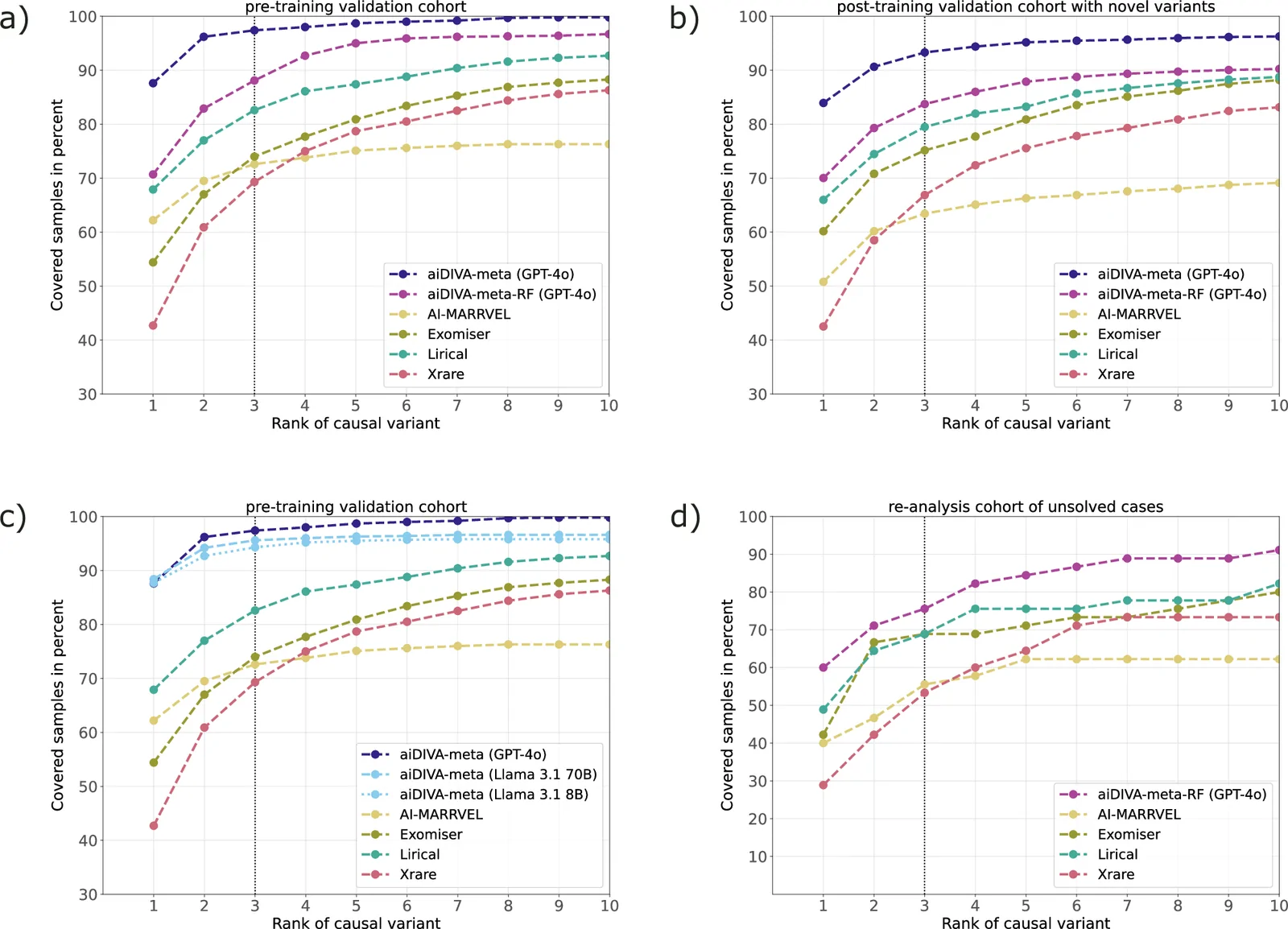
aiDIVA-meta performance. **a** Causal variant ranking on the pre-training validation cohort of 3041 containing variants present in ClinVar. The training of the aiDIVA-meta model was performed on 2/3 of our 3041 benchmark samples. This figure shows the ranking of the remaining 1/3 of the samples that were not used in the training. **b** Causal variant ranking on an independent set of 1014 cases sequenced and diagnosed after training of GPT-4o (June 2024) (“post-training validation cohort with novel variants”). **c** Causal variant ranking on the “pre-training validation cohort” showing the performance of two locally hosted LLMs (Llama 3.1 8B instruct and Llama 3.1 70B instruct) compared to GPT-4o. **d** Causal variant ranking of 45 newly solved patients that were previously unsolvable (“Re-analysis cohort”). Here we excluded the evidence-based model because it was initially used to prefilter the unsolved cases to find suitable cases for manual reanalysis.

To generate the final variant ranking suitable for clinical reporting, we developed an ensemble ML model named aiDIVA-meta, which integrates the outputs of all preceding models using the RF method (see Methods and Fig. 1). To obtain realistic performance estimates for both known pathogenic variants (i.e., already reported in ClinVar or similar databases), as well as for novel variants or variants of unknown significance, we trained two versions of the ensemble: one incorporating all preceding models (aiDIVA-meta) and another excluding the evidence-based models (aiDIVA-EB-dominant and aiDIVA-EB-recessive), referred to as aiDIVA-meta-RF. aiDIVA-meta and aiDIVA-meta-RF outperformed the individual ranking approaches (Supplementary Fig. 4). The aiDIVA-meta model (Fig. 4a, indigo curve) achieved the highest overall performance, ranking 97.4% of the causal variants among the top-3, and 88.1% of the causal variants at rank 1. Of note, aiDIVA-meta ranked significantly more causal variants at rank 1 compared to Exomiser, Lirical, AI-MARVEL, and Xrare. aiDIVA-meta-RF (purple line) significantly improved the results compared to aiDIVA-RF (Supplementary Fig. 4), despite excluding prior knowledge from clinical variant databases, underscoring the benefit of ensemble integration even without evidence-based features.

To evaluate the performance of aiDIVA-meta on cases diagnosed after LLM model training (June 2024 for GPT-4o) and caused by variants not in ClinVar or HGMD at the time of any model training, we collected an independent dataset of 1014 cases diagnostically solved between July 2024 and December 2025. We ensured that their causal variants were not present in ClinVar or HGMD prior to July 2024. We observe a minor reduction in aiDIVA performance, e.g. 97.4% vs. 93.3% of causal variants in top-3 ranks for aiDIVA-meta and 88.1% to 83.7% for aiDIVA-meta-RF (Fig. 4a vs. 4b). The maximum of identified causal variants within the top-10 ranks is reduced from 99.8% to 96.3% for aiDIVA-meta and from 96.7% to 90.1% for aiDIVA-meta-RF comparing the pre- and post-training cohorts.

In order to protect patient data and to include non-anonymized data, it would be preferred to use locally hosted LLMs instead of public instances such as GPT-4o. Therefore, we benchmarked the performance of aiDIVA-meta using two locally hosted LLMs instead of GPT-4o, namely Llama 3.1 8B and Llama 3.1 70B, both in the instruction-tuned version. aiDIVA-meta incorporating local LLMs is still outperforming all competing methods (Fig. 4c). We observe a minor decrease in performance compared to GPT-4o, which could likely be remedied by LLM fine-tuning or retrieval-augmented generation in the future.

### Re-analysis of previously unsolved cases

To evaluate the utility of aiDIVA for the automated re-analysis of previously unsolved cases, we revisited 4877 unsolved cases, originally evaluated using diagnostic-grade ES or GS prior to 2020. First, all cases were reannotated using the latest versions of relevant databases. Second, we prioritized 500 cases with the highest scoring top-ranked variants according to aiDIVA-EB, hypothesizing that newly available information (i.e., updated entries in ClinVar or HGMD) provides the greatest opportunity of solving previously unsolved cases. A genetics expert then reviewed the selected 500 cases and identified 45 cases that were now considered to be solved (based on an ACMG classification of (likely) pathogenic). Finally, we assessed the ranking of these newly identified causal variants using Exomiser, AI-MARRVEL, and aiDIVA-meta-RF. As none of these tools used knowledge from ClinVar or HGMD, their performance did not achieve 100% sensitivity, as expected (Fig. 4d). Nevertheless, aiDIVA-meta-RF strictly outperformed other tools, placing 41 of the 45 causal variants within the top-10 ranks (91.1%), including 34 variants in the top-3 (75.6%). These results support the use of aiDIVA for automated prioritization of previously unsolved cases that may now receive a firm diagnosis based on the incorporation of new knowledge and updated gene scores.

## Discussion

LLMs represent a novel and promising addition to the diagnostic toolbox for genome medicine. Despite limited benchmarking studies, our results demonstrate that LLMs meaningfully improve the prioritization of causal variants and genes and offer clinically relevant explanations for top candidates. However, naïvely including all coding variants into a prompt often leads to performance degradation due to information overload and irrelevant signals^41^. To mitigate this, we combined the LLM approach with conventional ML classifiers and weighted-sum models to pre-select plausible variants for LLM-based inferences. This ensemble strategy, implemented in the aiDIVA-meta model, ranked causal variants within the top-3 in 93.3% of expert-solved RD cases in the cohort collected post model training, with variants absent from both ClinVar and HGMD at the time of training. In the cohort collected prior to the model training date for which most variants had evidence in HGMD or ClinVar, the top-3 sensitivity increased to 97.4%. Finally, aiDIVA-meta-RF, which abstains from using any ClinVar or HGMD-derived information, achieved a sensitivity of 83.9%, reflecting the performance when prioritizing novel and de novo variants. These findings demonstrate the potential of aiDIVA-meta and aiDIVA-meta-RF to reduce the manual workload for clinical experts.

Although relatively rare, in-frame indels represent approximately 5% of all the pathogenic variants annotated in ClinVar. Generally, in-frame indels remain underrepresented in variant databases and are poorly supported by most functional impact predictors, as existing approaches for ML-based pathogenicity classification mostly ignore this variant type. In our study, we addressed this gap by representing in-frame indels as clusters of SNVs surrounding the affected region. This approximation significantly improved the classification performance, increasing the ROC AUC from 0.67 (CADD-Indel) to 0.85 (aiDIVA-RF). Nevertheless, the model’s performance on in-frame indels remained lower than that on SNVs, likely due to the limited training data and the inherent limitations of the SNV simulation strategy. Future improvements could include leveraging 3D protein structure modeling (e.g., with AlphaFold 3^42^) to estimate the functional effects of indels more accurately, although such integration was beyond the scope of this study.

Beyond initial diagnostics, aiDIVA also proved valuable for re-analysis of previously unsolved cases. Reanalysis workflows typically involve (a) periodic variant re-annotation, (b) scoring updates, and (c) identification of cases with a strong increase in the top-ranked score for expert review^3,43–45^. LLMs can accelerate this process by generating explanations for newly prioritized variants, supporting expert triage. We demonstrated this by integrating aiDIVA into the genome-diagnostics platform megSAP, performing automated re-annotation, re-scoring, re-ranking of variants, and prioritization of solvable cases. In our retrospective analysis of 4877 unsolved cases, aiDIVA preselected 500 high-confidence candidates based on updated scores, leading to 45 new diagnoses (approx. 1%) upon expert review. This demonstrates aiDIVA’s potential in large-scale automated reanalysis as variant databases evolve. Considering the rapid growth of genome datasets for RDs and still >60% unsolved cases, we expect a growing impact of the reanalysis model in clinical diagnostics.

While effective, LLM integration is not without limitations. Our benchmarking revealed that, in a small subset of cases, LLMs failed to reproduce answers consistently when the same prompt was repeated, indicating reduced reproducibility in borderline cases. Here, we investigated repeated prompting to evaluate variability in LLM responses. This reproducibility analysis on a small dataset of 200 cases showed an overall high reproducibility (5 out of 5 reproducible hits in up to 87% of cases) but also revealed 5% of cases with inconsistent responses. Hence, the reproducibility of the answers could be used as a quality measure prior to making decisions, although in routine clinical diagnostics the increased compute cost limits the usability of this approach. Moreover, new reasoning LLMs (for example GPT-o1^46^) can re-evaluate their own results, which is expected to increase the reproducibility of answers at the cost of longer computation times^47^.

We observed that including variant-level metadata, such as zygosity and functional impact, improved LLM performance in difficult cases. However, privacy concerns arise when sensitive patient data are included in prompts, especially when using cloud-based models. To address these concerns, one can either anonymize the information in the prompt (e.g., excluding variant positions) or set up local LLMs, which requires comprehensive expertise in AI. Moreover, LLMs often fail to recognize that a diagnostic query is unsolvable with current knowledge. We observed that LLMs provide unreliable answers for cases that remained without definite diagnosis based on expert review. As previously reported for LLMs of the GPT-4 family^48^, GPT-4o occasionally returns references to nonexistent or irrelevant literature, possibly contributing to wrongly prioritized genes. These limitations highlight the need for additional safety measures such as robust evaluation strategies or the integration of reasoning-enabled LLMs (e.g., GPT-o1), which may be able to reevaluate and validate their own outputs at the cost of increased computation time. Finally, structural variants, which account for a subset of genetic diagnoses, are not yet supported by aiDIVA, whereas Exomiser (since version 13.0.0) began to address this gap^49^.

Current LLMs are trained on a very broad range of data but are not tailored for clinical diagnostics. However, they can be specialized using techniques such as fine-tuning on domain-specific data. Fine-tuning allows the specialization of pre-trained open-source LLMs to a knowledge domain by changing the weights of the upper neuron layers or by adding an additional layer^50^. It is performed by providing prompts with correct answers for a knowledge domain. For instance, cases with their phenotypic description and a list of genes/variants are provided as prompts, and the name of the causal gene is provided as the answer. Prior studies suggest that fine-tuning with as few as 1000 domain-specific examples can yield substantial improvements in accuracy and contextual reasoning^51,52^. In addition, Retrieval Augmented Generation (RAGs) enables the embedding of external data sources, such as ClinVar or HPO, into the LLM^53^. Finally, refined strategies to pre-select the variants for LLM-inference could further improve the results, as would the inclusion of additional variant types, such as SVs. Ultimately, a switch to novel reasoning LLMs promises a strong gain in performance, reproducibility, and explainability of diagnostic AI.

While aiDIVA demonstrates robust performance and clinical utility, several avenues remain for future development:

- Integration with electronic health records and clinical feedback loops: A major opportunity lies in integrating aiDIVA with electronic health record (EHR) systems. Direct access to structured phenotype data, longitudinal clinical notes, and family histories could support real-time re-analysis and continuous diagnostic refinement. Additionally, establishing a feedback loop in which clinicians validate or adjust aiDIVA’s suggestions would enable adaptive learning over time. This “human-in-the-loop” approach could improve model accuracy and contextual understanding, fostering transparency in clinical settings.
- Federated learning and privacy-preserving genomic AI: Given the sensitive nature of genomic data, privacy-preserving methods such as federated learning present an attractive avenue for enhancing aiDIVA without compromising patient confidentiality. Federated learning enables decentralized model training across institutions, where only model parameters, and not patient data, are shared. This approach would allow aiDIVA to benefit from diverse, multi-ethnic datasets while complying with data protection regulations, ultimately improving model robustness and generalizability.
- Multi-omics integration for improved variant interpretation and newborn screening: Integrating multi-omics data into variant interpretation frameworks offers the potential to enhance diagnostic precision, particularly for variants of uncertain significance (VUS) or those located in non-coding or regulatory regions. Transcriptome data can help to identify aberrant splicing, allele-specific expression, or transcriptional dysregulation linked to specific variants. Likewise, proteomic and metabolomic data may provide downstream functional evidence, enabling better discrimination between benign and pathogenic alterations. This multi-layered evidence is especially relevant in newborn screening (NBS), where early detection of genetic disorders in still presymptomatic newborns is critical for timely intervention. As GS is increasingly being considered for use in NBS programs, AI-assisted interpretation tools, such as aiDIVA, will be essential to manage the large number of variants detected and provide clinically actionable insights.

In summary, aiDIVA provides AI models for assisting clinical experts with a highly sensitive and precise selection of potentially causal genetic variants in RDs. aiDIVA ranked the correct causal variant among the top-3 positions in 97.4% of the pre-training and 93.3% of the post-training cohort. In addition, aiDIVA also returns explanations for these suggestions, which could be utilized in downstream applications for clinical report generation, following expert inspection of the results.

## Methods

### Model overview

aiDIVA is a clinical decision support system for the classification, clinical interpretation and prioritization of likely clinically relevant variants observed in individuals affected with RDs. It comprises an ensemble of ML and statistical models, as shown in Fig. 1. aiDIVA performs the following subsequential tasks to get a final variant ranking.

- An RF model (aiDIVA-RF) is used to score and rank genetic variants according to their impact on protein function, potential pathogenicity, and fit to the respective phenotypes.
- An evidence-based ranking model (aiDIVA-EB) is applied, which relies on known evidence for variant pathogenicity found in publications and disease variant databases (ClinVar, HGMD, and OMIM, etc.). aiDIVA-EB provides two separate models for recessive and dominant inheritance (aiDIVA-EB-dominant and aiDIVA-EB-recessive). Both models are based on a scoring function utilizing known evidence, as well as molecular-genetic features and phenotypic characteristics of the respective individual (see Table 1 for a complete list).
- The ten highest-ranking variants of the models aiDIVA-RF, aiDIVA-EB-dominant, and aiDIVA-EB-recessive are used as inputs for the LLM GPT-4o. GPT-4o is used to refine the rankings and to generate explanations for the three most likely causal variants, including references to scientific literature.
- The top-10 variants of aiDIVA-RF, aiDIVA-EB-dominant, aiDIVA-EB-recessive, and the top-3 genes of their corresponding LLM refinement (LLM-aiDIVA-RF, LLM-aiDIVA-EB-dominant, and LLM-aiDIVA-EB-recessive) are combined as inputs for aiDIVA-meta, which creates the final pathogenicity ranking of an individual’s genetic variants.

An exemplary flowchart for variant annotation, pathogenicity ranking, and phenotype-based prioritization of the aiDIVA-RF model is provided in Supplementary Fig. 1.

### Generating a benchmarking set for diagnostic AIs

From 21,763 RD patients analyzed by GS or exome sequencing (ES) until 2023/06/01 at our institute, 4186 cases received a firm genetic diagnosis. From the solved cohort, we extracted a benchmark set for diagnostic AIs based on the following criteria: (1) definite diagnosis, (2) monogenic cause, (3) one or two causal SNVs or indels with ACMG^5^ classification: pathogenic or likely pathogenic variant(s), and (4) causal coding SNVs or indels with a population allele frequency of less than 1%. Compound heterozygotes were included if at least one of the two causal variants was a coding SNV or indel. Cases with pathogenic structural, deep intronic splicing or regulatory variants as well as repeat expansions were excluded from this benchmark dataset. This led to a final benchmark set of 3041 clinically solved RD cases matching all criteria.

### Solving the unsolved

To benchmark the ability of aiDIVA to increase the diagnostic yield via automated re-analysis, we used datasets from 4877 cases analyzed in 2020 or earlier and without a firm diagnosis. The data were re-analyzed, and variants were re-annotated using the latest version of the diagnostic NGS-pipeline megSAP^54^. Based on the aiDIVA-EB ranking, we then selected the top 500 cases based on the highest score for manual inspection by a clinical expert, resulting in 45 newly solved cases. These cases were used as a benchmark to test the ability of the AI models to solve unsolved cases.

### NGS analysis and variant annotation with megSAP and variant effect predictor

GS or ES data of clinical RD cases included in the benchmark sets described above were analyzed using the megSAP pipeline^54^, as described previously^1,35^. In brief, megSAP performs adapter trimming (SeqPurge), read alignment (BWA-mem2^55^), duplicate-flagging (SAMBLASTER^56^), indel-realignment (ABRA2^57^), and small variant calling (freebayes^58^). Part of the samples were analyzed using the Illumina DRAGEN^59^ (v4.3) mode of megSAP instead of the default pipeline described above. After variant calling, variants are annotated using a comprehensive in-house database combined with Variant Effect Predictor^39^. Variants were interpreted and classified according to the ACMG criteria^5^. Variant interpretation, variant filtering, and causal variant prioritization were performed by genetics experts and clinicians using the clinical decision support system GSvar^60^, as described previously^35^.

### ML-based pathogenicity estimation for single nucleotide variants

We trained an RF model (aiDIVA-RF) to score the pathogenicity of genetic variants using a diverse set of genomic annotations as features (Supplementary Table 4). aiDIVA-RF has been implemented in Python using the package scikit-learn^61^. Variants for training and testing of aiDIVA-RF were obtained from the ClinVar database (2023/07/17). ClinVar comprises variants annotated during clinical testing, ranging from benign to pathogenic variants, and provides the respective disease phenotype in case of pathogenic or likely pathogenic variants^12^. To generate binary labeled data, we classified (likely) benign variants as negative and (likely) pathogenic variants as positive. ClinVar variants also present in the in-house benchmark sets (see above) were removed from the training data to prevent biases in the evaluation and comparison with other models. Furthermore, we removed non-coding, synonymous coding, splicing, and frameshift indel variants from the training/testing set, while retaining non-synonymous coding SNVs and in-frame indels. Moreover, variants in low-confidence regions, as defined in the megSAP pipeline at https://github.com/imgag/megSAP/blob/master/data/misc/low_conf_regions.bed, were removed to improve the quality of the training data. Finally, we removed variants with a minor allele frequency (MAF) of >1% in gnomAD (v3.1.2).

The final ClinVar training dataset consisted of 90,400 (likely) pathogenic variants (SNVs: 87,750; in-frame indels: 2650) and 65,202 (likely) benign variants (SNVs: 63,900; in-frame indels: 1302). The dataset was split in a 90:10 ratio into independent training and testing datasets.

To investigate the generalizability of the trained RF model, we created a second test set using variants from the Human Gene Mutation Database (HGMD) (PRO_2022_04) not overlapping with ClinVar. Since HGMD only contains (likely) pathogenic variants, we included random presumed benign variants from the gnomAD database (v3.1.2) with MAFs ranging from 0.1% to 1%, matching the upper limit in HGMD. We applied the same filters to the HGMD test data as described for the ClinVar data.

We used VEP to annotate all variants with various features (Supplementary Table 2), including maximum MAF, functional impact, evolutionary conservation, and loss-of-function intolerance. Missing features are imputed with the median value taken from the annotation source, except missing allele frequency, as well as damage scores only available for missense variants. For variants absent from gnomAD, the MAF was set to 0. High-impact loss-of-function variants (e.g., stop-gains) not scored by AlphaMissense and REVEL are set to a pathogenicity score of 1 instead of the median. aiDIVA-RF was then trained using the ClinVar training set with binary labels (benign and pathogenic). The parameters used for training are listed in Supplementary Table 5. These parameters were selected via a grid search with 10-fold cross-validation.

### ML-based pathogenicity estimation for small insertions and deletions

The annotation of in-frame indels remains difficult because they are not scored at all by many pathogenicity predictors or because pre-calculated scores are missing for them. To overcome this annotation issue, we converted both deletions and insertions into a cluster of subsequent SNVs, starting 3nt upstream and ending 3nt downstream of the indel. Positions modified in this manner were assigned a random nucleotide substitution. For deletions, all nucleotides along the deleted sequence and 3nt upstream/downstream were modified, whereas for insertions only the 3nt upstream and downstream were changed. Our procedure assumes that in-frame deletions and insertions affect their immediate neighborhood and surrounding regions but have a negligible effect on other regions of the protein. This strategy enabled the annotation of indels using the same feature set applied to SNVs. For each annotation feature, we computed the mean value across all random substitutions spanning the indel region. Missing values are imputed as described for SNVs above. To incorporate in-frame indels in the RF model utilized in aiDIVA-RF, we added 2650 pathogenic and 1302 benign indels from ClinVar—using a 90:10 split for training and testing.

Frameshift indels are not included in RF training or scoring. Instead, frameshift indels are considered loss-of-function variants and set to a pathogenicity score of 0.9. Compared to LoF-SNVs, we reduced the pathogenicity score (1.0 vs. 0.9) due to the increased fraction of false positive indel calls observed for most variant detection algorithms.

### Prioritization of causal disease variants using pathogenicity scores and phenotype matching

Based on the predicted pathogenicity scores for non-synonymous SNVs and in-frame indels provided by our trained RF model, we prioritized the most likely disease-causing variants using a ranking approach. Splice-variants were included in the ranking using precomputed SpliceAI scores. In addition to pathogenicity scores, aiDIVA-RF uses HPO terms^27^ to score the similarity between phenotypes associated with a candidate gene and the respective disease phenotypes. Gene-associated HPO terms were derived from the HPO database (https://hpo.jax.org/, v2023-04-05). We used the Lin similarity measure to calculate the node-based semantic similarity between the two HPO term sets (gene-HPO set vs. patient-HPO set) within the hierarchical tree structure of the HPO ontology, as previously described^62^. The result of this calculation was used as a phenotype similarity score, indicating how well the patient’s phenotype matched a certain gene affected by a variant.

A second phenotype similarity score was computed by considering neighboring genes of the candidate disease gene based on protein-protein interaction information from STRING^63^ (v11.0b). If a gene interacting with the gene of interest had a high phenotypic similarity to the patient, calculated as described above, we assumed that the gene of interest may be an unknown cause of the disease.

A weighted sum approach was applied to combine the pathogenicity score, phenotype similarity, and gene network phenotype similarity using 2/3 of the solved in-house cases to train the weights. The training tested all weight combinations in 0.1 steps to minimize the rank of causal variants. To reduce the number of reported disease-variant candidates, variants with an aiDIVA-RF pathogenicity score below 0.7, which are also of low confidence, low complexity, or in repetitive regions, were removed. In addition, variants with a Lin score of zero for both phenotype similarity measures were excluded. Supplementary Table 3 shows an overview of all filters that are applied during the prioritization step of aiDIVA-RF. Supplementary Table 1 shows all supported variant consequences according to the Ensembl variation.

### Evidence-based ranking

The evidence-based model aiDIVA-EB is a weighted sum-based ranking method developed for the clinical decision support system GSvar, which is routinely used for genome diagnostics. The input of aiDIVA-EB is an annotated list of variants and HPO-encoded individual phenotypes of a patient. Evidence-based ranking was conducted in two stages: (1) the exome/genome variant list was prefiltered to remove unlikely candidates; (2) the remaining variants were scored and ranked based on predefined weighted criteria.

- We applied several filters: First, all variants with a MAF > 1% in gnomAD were excluded. Next, only variants in protein-coding and splicing regions were included, i.e., with a VEP impact of HIGH, MODERATE, or LOW. To remove pipeline-specific artifacts, we discarded variants that occurred more than ten times with the same genotype in an in-house database. Finally, variants with a base quality of less than 30 or a mapping quality of less than 20 were removed. After filtering, only rare coding and splicing variants unlikely to represent sequencing artifacts were retained. While some pathogenic variants have been reported in intronic and intergenic regions, these would typically be excluded by standard filtering criteria. To address this limitation, we retained variants that met the following criteria: (i) were described as (likely) pathogenic in ClinVar (2024/08/05) or HGMD (PRO_2024_02) or that were predicted to affect splicing (SpliceAI > 0.5), and (ii) had MAF < 1%. Typically, 200–500 variants remained after filtering.
- These variants were scored using a point-based system, with separate models for dominant (aiDIVA-EB-dominant) and recessive (aiDIVA-EB-recessive) inheritance. Within each model, criteria were assessed independently, and points were assigned and summed up. Finally, the variants were ranked according to their overall scores in descending order. Table 1 outlines the evidence criteria and corresponding point assignments for both aiDIVA-EB-dominant and aiDIVA-EB-recessive models.

### LLM model

We used the LLM GPT-4o^32^ developed by OpenAI to refine the rankings generated by aiDIVA-RF and aiDIVA-EB, and to generate explanations for the suggested causal gene variants. We integrated GPT-4o into aiDIVA using the OpenAI RESTful API (https://platform.openai.com/docs/api-reference, accessed: 2025/05/23). For all analyses presented in this manuscript, we used the snapshot *gpt-4o-2024-05-13* of the GPT-4o model with a temperature of 0.1. Briefly, we first selected the top 10 ranked genes from each of the aiDIVA-RF, aiDIVA-EB-dominant, and aiDIVA-EB-recessive models as inputs for three separate queries (LLM-aiDIVA-RF, LLM-aiDIVA-EB-dominant, and LLM-aiDIVA-EB-recessive). Furthermore, each variant was assigned to one of five impact types (protein truncating, missense, in-frame insertion, in-frame deletion, and splice region). If the variant was present on both alleles, we added the keyword “homozygous”. In addition, we used the recorded disease phenotypes, age at diagnosis, and sex as characteristics to describe a case to GPT-4o. Combining this data, we automatically generated the following prompt for the respective individual in our in-house cohort (see also the Supplementary Section *Prompt Scheme*):

*“A [sex] rare disease patient of age [age] has the following symptoms: [list of phenotypes as HPO terms]. A causal variant in which of the following candidate genes would best explain these symptoms? For each candidate gene the consequence of the variant is given in brackets. Furthermore, homozygous variants are labeled in brackets. Candidate genes:*

*Gene-1 (loss-of-function variant)*

*Gene-2 (homozygous missense variant)*

*Gene-3 (splice region variant)*

*…*

*Gene-10 (homozygous inframe insertion/deletion)”*

To perform the analyses, we initialized the API using a comprehensive set of instructions (Supplementary Section *System Instructions*). Each query consisted of instructions and prompts (diagnostic questions) for five patients. Each of the five prompts was created according to the schema described above. As a response, we obtained a table according to the schema described in the system instructions given with each request. We requested reasoning from GPT-4o for choosing the 1st, 2nd, and 3rd ranked genes.

The data shared with GPT-4o is considered anonymized, as no variant positions and only 10 affected genes plus the patient’s phenotype, sex, and age were shared. Use of the public GPT-4o was approved by the data protection officer of the University Hospital of Tübingen.

### aiDIVA-meta

The aiDIVA-meta model is an ensemble of all ML and statistical methods presented above, which generates the final candidate disease variant/gene ranking. aiDIVA-meta again utilizes an RF model that was trained using the ranks and scores of all approaches (rankings generated by aiDIVA-RF, aiDIVA-EB-dominant, and aiDIVA-EB-recessive, LLM-aiDIVA-RF, LLM-aiDIVA-EB-dominant, and LLM-aiDIVA-EB-recessive) as features (Supplementary Table 6). The originating EB model is specified through the two binary features “dominant” and “recessive”. To train aiDIVA-meta, we performed a random split using 2/3 of our in-house benchmark set of 3041 clinically solved Mendelian disease cases as training data and tested its performance on the remaining 1/3 of the cases. The training parameters are shown in Supplementary Table 7. The features and training parameters used for the aiDIVA-meta-RF model are shown in Supplementary Table 8 and Supplementary Table 9.

### Benchmarking Exomiser

Exomiser is a variant prioritization tool used to rank candidate variants in patients with RDs^22^. It incorporates different pathogenicity scores and a random walk-based approach to measure the association between a gene and an individual’s disease phenotypes. For benchmarking, we used Exomiser v13.2.1 with the recommended default parameters, applying the hiphive prioritiser in “human-only” mode. To achieve ideal benchmark conditions, we also applied the recommended inheritance-specific frequency filters. All parameters and settings applied are described in the Supplementary Section *Exomiser*. A detailed documentation of Exomiser usage is available at https://exomiser.readthedocs.io/en/13.2.1/ (accessed: 2025/05/23).

### Benchmarking AI-MARRVEL

AI-MARRVEL is an ML method for prioritizing causal variants in individuals with RDs^21^. Similar to aiDIVA and Exomiser, AI-MARRVEL integrates genomic and phenotypic information of affected individuals in their model. We used AI-MARRVEL v1.0.1 (https://github.com/LiuzLab/AI_MARRVEL, accessed: 2024/07/31) and ran the AI-MARRVEL Docker image with default settings to generate all benchmark results.

### Benchmarking Lirical

Lirical predicts a patient’s disease based on phenotype information with the option to include genotyping information^26^. We used Lirical v2.4.0 in the genotype-enabled mode, where Lirical reports the predicted disease and the ranked potential causal variants. We ran Lirical through the command line interface in “prioritize” mode as described in the documentation (https://thejacksonlaboratory.github.io/LIRICAL/stable/running.html#prioritize-run-lirical-with-via-cli-options) using phenotype and variant data originally prepared for Exomiser. Furthermore, we included the sex and age information given for each patient.

### Benchmarking Xrare

Xrare is an ML-based method to prioritize causal variants combining phenotype and genotype evidence^25^. Xrare uses a phenotype similarity scoring method called Emission-Reception Information Content (ERIC) that is combined with medical domain knowledge based on ACMG criteria. We used the Docker container xrare37-pub v2021 available through the Xrare website (https://web.stanford.edu/group/wonglab/Xrare/xrare-pub.2021.html) and ran Xrare as described in the documentation, providing a VCF file and a list of HPO terms. Since the annotation data provided in the Docker container is only available for the hg19 reference genome, we lifted the variant coordinates of our patient samples from hg38 to hg19.

### Ethics statement

Written informed consent was obtained from all individuals or their guardians and archived. All procedures were performed in accordance with the Helsinki Declaration. Individual-level data were de-identified. The study was approved by the ethics committee of the medical faculty and the local Institutional Review Board of the Medical Faculty of the University of Tübingen, Germany (066/2021BO2).

## Supplementary information


Supplementary information


## Data Availability

Training data used to train the aiDIVA-RF model have been obtained from ClinVar. Causal variants used for model evaluation in this study have been uploaded to ClinVar using the following submitter: Institute of Medical Genetics and Applied Genomics (University Hospital Tübingen). Additional data are available from the corresponding author upon individual request. Patient data not included may be subject to patient confidentiality. Raw sequencing data were not consented for sharing.

## Code availability

The aiDIVA source code, along with the three trained random forest models (aiDIVA-RF, aiDIVA-meta, and aiDIVA-meta-RF), is available on GitHub at https://github.com/imgag/aiDIVA and through the Zenodo record at https://zenodo.org/records/16966353. The two aiDIVA-EB models can be accessed via GSvar and through the command line tool VariantRanking (https://github.com/imgag/ngs-bits/blob/master/doc/tools/VariantRanking/index.md), with model names GSvar_v2_dominant and GSvar_v2_recessive. The source code for this tool is available on GitHub at https://github.com/imgag/ngs-bits and through the Zenodo record at https://zenodo.org/records/15051584.
